# A Comparative Study of Transforaminal Epidural Steroid Injection (TFESI) Versus Caudal Epidural Steroid Injections (CESI) in the Management of Lumbar Radiculopathy

**DOI:** 10.7759/cureus.85582

**Published:** 2025-06-08

**Authors:** Nidhil Noushad CP, Hariprasad Seenappa, Nagakumar JS, Gils Thampi

**Affiliations:** 1 Department of Orthopaedics, Sri Devaraj Urs Medical College, Sri Devaraj Urs Academy of Higher Education and Research, Kolar, IND; 2 Department of Orthopaedics, Sri Devaraj Urs Academy of Higher Education and Research, Kolar, IND; 3 Department of Orthopaedics, Sri Devaraj Urs Medical College, Sri Devraj Urs Academy of Higher Education and Research, Kolar, IND

**Keywords:** caudal epidural steroid injection, cori, low back pain, lumbar radiculopathy, modi, mrm, pain management, transforaminal epidural injection, vas

## Abstract

Background

Lumbar radiculopathy is a common orthopedic condition often managed non-surgically with epidural steroid injections (ESIs). Among the three commonly used approaches - caudal, interlaminar, and transforaminal - the ideal route for maximum efficacy remains under debate. This study compares the effectiveness of caudal versus transforaminal ESI in terms of pain relief, functional improvement, and disability reduction over a 6-month follow-up period.

Methodology

A retrospective cross-sectional study was conducted at R.L. Jalappa Hospital and Research Center and Sri Devraj Urs Medical College, Karnataka, India, from June 2021 to June 2024. Sixty patients with confirmed lumbar radiculopathy and radiological evidence of disc prolapse were divided into two groups: transforaminal (n=30) and caudal (n=30). All participants had failed six weeks of conservative management prior to intervention. Outcomes were assessed at baseline and 1, 3, and 6 months post-intervention using the Visual Analog Scale (VAS), Modified Oswestry Disability Index (MODI), Clinical Outcome Rating Index (CORI), and Modified Roland-Morris (MRM) scores. Statistical analysis included independent t-tests, repeated measures ANOVA, and descriptive statistics with significance set at p < 0.05.

Results

The mean age of participants was 43.5 ± 8.33 years in the transforaminal (TF) group and 40.2 ± 8.96 years in the caudal group (p = 0.143). The sex distribution exhibited slight variation, with a predominance of males in the TF group (n = 17, 56.7%) compared to a predominance of females in the caudal group (n = 16, 53.3%); however, this difference was not statistically significant (p = 0.432). Both groups demonstrated significant improvements in all outcome measures over time (p < 0.001). The transforaminal group (n = 30) exhibited superior short-term outcomes for 1 month, with lower VAS scores (3.87 vs 5.13) and MODI scores (35.47% (n = 30) vs 43.4% (n = 30)). At 3 months, the caudal group began showing better improvement. By 6 months, the caudal group demonstrated significantly better outcomes across all parameters: lower VAS scores (1.47 vs 2.13, p < 0.001), reduced MODI scores (11.5% (n = 30) vs 18.13% (n = 30), p < 0.001), improved CORI scores (1.23 vs 3.88), and higher MRM percentages (86.6% (n = 30) vs 67.95% (n = 30), p < 0.001). Repeated measures ANOVA confirmed significant time × group interactions for VAS (F = 49.2, p < 0.001), MODI (F = 59.2, p < 0.001), CORI (F = 2.7, p < 0.001), and MRM scores (F = 173.8, p < 0.001), indicating different recovery trajectories between the two groups. These findings suggest that while transforaminal injections provide more immediate relief, caudal injections offer superior long-term efficacy for managing lumbar radiculopathy.

Conclusions

While transforaminal ESIs offer faster short-term relief, caudal ESIs demonstrated superior long-term outcomes in terms of both pain reduction and functional recovery. Caudal ESIs, being technically simpler and safer, may be a favorable alternative for long-term management in lumbar radiculopathy.

## Introduction

Lumbar radiculopathy, characterized by lower back discomfort that radiates to lower extremities, significantly impacts quality of life and functionality among affected individuals. It frequently results from compression of the nerve roots as a result of disc herniation, degenerative disc disease, or spinal stenosis [1,2]. With a lifetime prevalence of 80% and an annual prevalence as high as 45%, low back pain (LBP) and associated radicular symptoms pose major clinical and socio-economic challenges worldwide [3].

Various conservative treatments are available for managing lumbar radiculopathy, including physical therapy, pharmacological management, and epidural steroid injections (ESIs). Epidural steroid injections are a frequently utilized non-surgical intervention, primarily aiming at pain relief and improvement in function and targeted reduction of inflammation in affected nerve roots [4,5]. ESIs can be delivered via multiple anatomical approaches, including transforaminal (TF), caudal, and interlaminar routes, each having specific advantages and limitations [6].

The TF approach, considered more targeted, delivers medication directly near the affected nerve root with relatively smaller medication volumes, offering precise localization and potentially superior immediate relief [7]. However, this method involves relatively higher procedural complexity, longer duration, and radiation exposure from fluoroscopy [8]. Conversely, caudal epidural steroid injection (CESI), being less technically demanding and safer, is favoured for ease of administration and fewer complications, albeit requiring larger volumes of medication with less precise targeting [9].

In the literature, there is continuous discussion regarding the comparative efficacy of TF and caudal routes. Recent systematic reviews and meta-analyses suggest both approaches effectively reduce pain and improve functional scores in the short term, yet long-term outcomes and comparative efficacy remain inconclusive [10-12]. Some studies have favoured TF injections for their specificity, while others highlighted the caudal route for its safety profile, simplicity, and efficacy in providing sustained symptomatic relief [13-16].

Despite numerous studies examining these techniques individually, there remains insufficient comparative evidence specifically addressing their relative effectiveness in different clinical contexts or across various follow-up durations [17,18]. Given this ambiguity and the significant clinical implications of effectively managing lumbar radiculopathy, further comparative research is crucial.

The study aims to make a comparison in terms of the efficacy of transforaminal versus caudal epidural steroid injections for managing lumbar radiculopathy, focusing specifically on short-term and long-term outcomes in pain relief, functional disability decline, and overall clinical improvement. Our objective is to determine whether either of these techniques demonstrates superiority in clinical practice, thus aiding clinicians in informed decision-making tailored to patient-specific needs.

## Materials and methods

Study design and setting

A retrospective cross-sectional analytical study was conducted to evaluate and compare the effectiveness of caudal versus transforaminal epidural steroid injections in patients with lumbar radiculopathy. The study was conducted at the Department of Orthopedics, R.L. Jalappa Hospital and Research Center, affiliated with Sri Devaraj Urs Medical College, Kolar, Karnataka, India.

Study period

The study encompassed patients who underwent epidural steroid injections for lumbar radiculopathy between June 2021 and June 2024.

Ethics committee approval

Prior to commencement, the study protocol received approval from the Central Ethics Committee, Research and Development Cell, Sri Devraj Urs Academy of Higher Education and Research (Approval No: SDUMC-PG/25 /NE/-2025-26). The study was conducted in accordance with the ethical standards outlined in the Declaration of Helsinki. All patient data were anonymized, and confidentiality was maintained throughout the research process.

Inclusion criteria

The study included adult patients aged between 18 and 75 years who presented with clinically diagnosed lumbar radiculopathy characterized by radiating pain in a specific dermatomal distribution. All participants had radiological evidence (MRI) of lumbar disc herniation that correlated with their clinical symptoms and had undergone either transforaminal or caudal epidural steroid injection after failing at least six weeks of conservative management. Only patients with complete follow-up data at 1, 3, and 6 months post-intervention and who provided informed consent for participation were included in the final analysis.

Exclusion criteria

The study excluded patients with a history of previous lumbar spine surgeries to avoid confounding factors related to post-surgical changes. Patients on concurrent anticoagulant therapy or with coagulopathies were excluded due to increased risk of procedural complications. Those with known allergies to contrast media, steroids, or local anesthetic agents were also excluded for safety considerations. Additional exclusion criteria comprised patients with significant local infection at the injection site or underlying spinal infections, unstable spinal conditions or fractures requiring surgical intervention, pregnant or breastfeeding women, those who had received epidural steroid injections within the previous six months, patients with incomplete follow-up data, and individuals with comorbid conditions such as uncontrolled diabetes, immunosuppressive disorders, or malignancy that might influence treatment outcomes or interpretation.

Sample size estimation

The sample size was calculated using the following formula:

$N = \frac{[Z_{1-\alpha/2} + Z_{1-\beta}]^2 \times 2 \times \sigma^2}{(\mu_1 - \mu_2)^2}$

Where:

$Z_{1-\alpha/2}$ = 1.96 (two-tailed probability for 95% confidence interval)

$Z_{1-\beta}$ = 0.84 (two-tailed probability for 80% power)

$\mu_1$ = 71.36 (mean of Peak pronation strength in recovered group)

$\mu_2$ = 72.51 (mean of Peak pronation strength in unrecovered group)

Based on the previous study by Mendoza-Lattes et al. (2009), which compared the effectiveness of caudal versus transforaminal epidural steroid injections in 30 patients with lumbosacral radicular pain, reporting mean VAS scores of 3.2 ± 1.8 and 4.7 ± 2.1, respectively [19]. Using the standard formula for sample size calculation with a 95% confidence interval and 80% power, the minimum required sample size was calculated to be 28 patients per group. To account for potential loss to follow-up (approximately 10%), a total of 30 patients were included in each group, resulting in a total sample size of 60 patients.

Sampling method

Consecutive sampling technique was employed to enroll eligible patients who underwent either transforaminal or caudal epidural steroid injections during the study period. Patients were allocated to the respective groups based on the intervention they received as determined by their treating physician. The decision for the type of epidural approach was made by the treating orthopedic surgeon based on clinical presentation, imaging findings, and patient-specific factors.

Data collection procedure

A standardized data collection proforma was developed to extract relevant demographic, clinical, and outcome data from medical records. Demographic variables included age, sex, occupation, duration of symptoms, and distribution of radiculopathy (bilateral, left, right, or isolated). Clinical variables included presenting symptoms, neurological deficits, and radiological findings.

Pain intensity was assessed using the Visual Analog Scale (VAS), which is a validated 10-point scale with 0 representing no pain and 10 representing the worst possible pain. Functional disability was evaluated using the Modified Oswestry Disability Index (MODI), which quantifies disability as a percentage, with higher percentages indicating greater disability. The Clinical Outcome Rating Index (CORI) and Modified Roland-Morris (MRM) scores were also collected to comprehensively assess clinical outcomes and functional recovery.

These measurements were recorded at baseline (pre-intervention) and at follow-up visits scheduled at 1, 3, and 6 months post-intervention. All assessments were conducted by trained orthopedic residents who were blinded to the type of intervention received by the patients to minimize assessment bias.

Intervention procedures

Transforaminal Epidural Steroid Injection (TFESI)

Patients were positioned prone on the fluoroscopy table with a pillow under the abdomen to reduce lumbar lordosis. After sterile preparation and draping, the target level was identified under fluoroscopic guidance. Local anesthesia (2% lidocaine) was administered to the skin and subcutaneous tissues. A 22-gauge spinal needle was advanced using a subpedicular approach to target the affected nerve root. Correct needle placement was confirmed with the injection of 0.5-1 ml non-ionic contrast medium (Iohexol 300 mg/ml) and visualization of characteristic perineural contrast flow. Following confirmation, a mixture of 2 ml methylprednisolone (80 mg) combined with 1 ml bupivacaine (0.5%) and diluted with 1 ml normal saline was administered.

Caudal Epidural Steroid Injection (CESI)

Patients were positioned prone with a pillow under the pelvis. The sacral hiatus was identified by palpation and confirmed under fluoroscopic guidance. After sterile preparation and draping, local anesthesia (2% lidocaine) was administered to the skin and subcutaneous tissues. A 22-gauge needle was introduced into the epidural space through the sacral hiatus at a 45-degree angle and advanced under fluoroscopic guidance. Proper placement was confirmed using 2-3 ml of non-ionic contrast medium (Iohexol 300 mg/ml) to visualize the characteristic epidural spread. Following confirmation, a mixture of 2 ml methylprednisolone (80 mg) combined with 2 ml lignocaine (2%), diluted with 6 ml normal saline, was administered.

All procedures were performed by experienced orthopedic surgeons with specialized training in spine interventions, under strict aseptic conditions in a dedicated procedure room equipped with fluoroscopy facilities.

Study tools

The study employed the following tools:

Visual Analog Scale (VAS): A 10-point scale used to quantify pain intensity.

Modified Oswestry Disability Index (MODI): A validated questionnaire consisting of ten sections, each scored from 0 to 5, measuring functional disability in activities of daily living. The cumulative score is expressed as a percentage of disability.

Clinical Outcome Rating Index (CORI): A composite index evaluating clinical outcomes based on pain relief, analgesic consumption, and return to activities.

Modified Roland-Morris (MRM): A validated tool measuring functional recovery and motor improvement, expressed as a percentage.

Data analysis

Statistical analysis was performed using IBM SPSS Statistics (version 25.0) for Windows (IBM Corp., Armonk, USA). Descriptive statistics were calculated for demographic and clinical characteristics, with continuous variables expressed as mean ± standard deviation and categorical variables as frequencies and percentages. Comparisons between the transforaminal and caudal groups were performed using independent samples t-tests for continuous variables. Repeated measures ANOVA with Greenhouse-Geisser correction was employed to analyze changes in outcome parameters over time within and between groups. Post hoc analyses with Bonferroni correction were conducted for multiple comparisons. The significance level was set at p < 0.05 for all statistical tests. Effect sizes were calculated using Cohen's d, with values of 0.2, 0.5, and 0.8 considered small, medium, and large effects, respectively.

## Results

The baseline characteristics demonstrated comparable demographic and clinical profiles between intervention groups (Table 1). The mean age of participants was 43.5 ± 8.33 years in the transforaminal (TF) group and 40.2 ± 8.96 years in the caudal group (p = 0.143). The sex distribution exhibited slight variation, with a predominance of males in the TF group (17, 56.7%) and a predominance of females in the caudal group (16, 53.3%); however, this difference was not statistically significant (p = 0.432). The patterns of radiculopathy were similar in both groups, with eight (26.7%) TF patients and 13 (43.3%) caudal patients having symptoms on both sides, 11 (36.7%) TF patients and six (20.0%) caudal patients having symptoms on the left side, and 11 (36.7%) TF patients and 10 (33.3%) caudal patients having symptoms on the right side. Only one patient (3.3%) in the caudal group presented with isolated radiculopathy. These findings confirm adequate baseline comparability between intervention groups, supporting valid subsequent outcome comparisons.

**Table 1:** Baseline characteristics of study participants (N = 60) P-values calculated using independent t-test for continuous variables and chi-square test for categorical variables.

Variable	Transforaminal Group (n = 30)	Caudal Group (n = 30)	Total (N = 60)	p-value
Age (Mean ± SD), years	43.5 ± 8.33	40.2 ± 8.96	41.9 ± 8.74	0.143
Male	17	14	31	0.432
Female	13	16	29	
Radiculopathy - Bilateral	8	13	21	0.196
Radiculopathy - Left (L)	11	6	17	
Radiculopathy - Right (R)	11	10	21	
Radiculopathy - Isolated (I)	0	1	1	

Pain intensity assessment using the Visual Analog Scale (VAS) demonstrated temporal evolution with significant between-group differences (Table 2). At baseline, both groups reported comparable pain severity (TF: 6.83 ± 0.531, Caudal: 6.6 ± 0.563; p = 0.104). At 1-month follow-up, the TF group exhibited superior early pain reduction (3.87 ± 0.629) compared to the caudal group (5.13 ± 0.681; p < 0.001), with a mean difference of -1.26 (95% CI: -1.61, -0.92). This advantage persisted at 3 months, with the TF group maintaining lower pain scores (2.73 ± 0.45 vs. 3.23 ± 0.568; p < 0.001). However, by the 6-month assessment, a significant reversal occurred, with the caudal group demonstrating superior sustained pain relief (1.47 ± 0.507) versus the TF group (2.13 ± 0.507; p < 0.001), suggesting differential temporal efficacy profiles between techniques.

**Table 2:** Longitudinal assessment of visual analog scale scores in patients receiving transforaminal versus caudal epidural steroid injections (N = 60) Values presented as Mean ± SD; ^*^statistically significant at p < 0.05

Timepoint	Transforaminal Group (n = 30)	Caudal Group (n = 30)	Mean Difference (95% CI)	p-value
Baseline	6.83 ± 0.531	6.6 ± 0.563	0.23 (-0.05, 0.51)	0.104
1 month	3.87 ± 0.629	5.13 ± 0.681	-1.26 (-1.61, -0.92)	<0.001^*^
3 months	2.73 ± 0.45	3.23 ± 0.568	-0.50 (-0.76, -0.24)	<0.001^*^
6 months	2.13 ± 0.507	1.47 ± 0.507	0.66 (0.40, 0.92)	<0.001^*^

Functional disability assessment through the Modified Oswestry Disability Index (MODI) revealed dynamic patterns of recovery (Table 3). Baseline disability levels were comparable between groups (TF: 56.43% (n = 30); Caudal: 54.93% (n = 30); p = 0.209). At the 1-month evaluation, the TF group exhibited significantly greater functional improvement (35.47% (n = 30)) compared to the caudal group (43.4% (n = 30); p < 0.001), with a mean difference of -7.93% (95% CI: -10.16, -5.70). However, by 3 months, the pattern reversed, with the caudal group demonstrating superior functional recovery (20.07% (n = 30)) versus the TF group (26.4% (n = 30; p < 0.001). This advantage for the caudal approach became more pronounced at 6 months (11.5% (n = 30) vs. 18.13% (n = 30); p < 0.001), suggesting potentially different mechanisms of action affecting the trajectory of functional improvement between intervention techniques.

**Table 3:** Temporal evaluation of Modified Oswestry Disability Index (MODI) in patients with lumbar radiculopathy following different epidural injection approaches (N = 60) Values presented as Mean ± SD; MODI scores expressed as percentage of disability; ^*^statistically significant at p < 0.05

Timepoint	Transforaminal Group (n = 30)	Caudal Group (n = 30)	Mean Difference (95% CI)	p-value
Baseline	56.43 ± 4.057	54.93 ± 5.044	1.50 (-0.85, 3.85)	0.209
1 month	35.47 ± 4.377	43.4 ± 4.166	-7.93 (-10.16, -5.70)	<0.001^*^
3 months	26.4 ± 5.069	20.07 ± 3.463	6.33 (4.09, 8.57)	<0.001^*^
6 months	18.13 ± 3.037	11.5 ± 1.456	6.63 (5.42, 7.84)	<0.001^*^

Clinical Outcome Rating Index (CORI) scores demonstrated temporal evolution with intervention-specific patterns (Table 4). At baseline and 1 month, no significant differences were observed between groups (baseline: p = 0.106; 1 month: p = 0.133). By the 3-month assessment, the caudal group exhibited significantly better clinical outcomes (3.34 ± 0.802) compared to the TF group (4.36 ± 1.066; p < 0.001). This advantage became more pronounced at 6 months, with substantially lower CORI scores in the caudal group (1.23 ± 0.430) versus the TF group (3.88 ± 0.958; p < 0.001), representing a mean difference of 2.65 (95% CI: 2.29, 3.01). This pattern suggests superior sustained clinical benefit with the caudal approach despite similar early outcomes.

**Table 4:** Longitudinal assessment of Clinical Outcome Rating Index (CORI) scores following transforaminal versus caudal epidural steroid injections (N = 60) Values presented as Mean ± SD; ^*^statistically significant at p < 0.05

Timepoint	Transforaminal Group (n = 30)	Caudal Group (n = 30)	Mean Difference (95% CI)	p-value
Baseline	7.24 ± 1.878	6.58 ± 1.172	0.66 (-0.14, 1.46)	0.106
1 month	4.88 ± 1.338	5.33 ± 0.993	-0.45 (-1.04, 0.14)	0.133
3 months	4.36 ± 1.066	3.34 ± 0.802	1.02 (0.55, 1.49)	<0.001^*^
6 months	3.88 ± 0.958	1.23 ± 0.430	2.65 (2.29, 3.01)	<0.001^*^

Motor recovery assessment through Modified Roland-Morris (MRM) scores revealed significant between-group differences throughout the evaluation period (Table 5). Notably, baseline MRM scores differed significantly between groups (TF: 25.88% (n = 30); Caudal: 19.68% (n = 30); p = 0.019), representing a methodological limitation requiring consideration in interpretation. At the 1-month evaluation, the TF group demonstrated markedly superior motor recovery (50.99% (n = 30)) compared to the caudal group (24.14% (n = 30); p < 0.001), with a substantial mean difference of 26.85% (95% CI: 22.47, 31.23). By 3 months, both groups showed considerable improvement, though the TF group maintained a modest statistical advantage (61.39% (n = 30) vs. 58.22% (n = 30); p = 0.035). However, at the 6-month assessment, a dramatic reversal occurred, with the caudal group achieving significantly superior long-term motor recovery (86.6% (n = 30)) compared to the TF group (67.95% (n = 30); p < 0.001), demonstrating a mean difference of -18.65% (95% CI: -21.13, -16.17).

**Table 5:** Temporal changes in Modified Roland-Morris (MRM) scores following different epidural steroid injection techniques for lumbar radiculopathy (N = 60) Values presented as Mean ± SD; MRM scores expressed as percentage of recovery; ^*^statistically significant at p < 0.05

Timepoint	Transforaminal Group (n = 30)	Caudal Group (n = 30)	Mean Difference (95% CI)	p-value
Baseline	25.88 ± 13.367	19.68 ± 4.179	6.20 (1.05, 11.35)	0.019^*^
1 month	50.99 ± 10.623	24.14 ± 5.573	26.85 (22.47, 31.23)	<0.001^*^
3 months	61.39 ± 7.164	58.22 ± 3.646	3.17 (0.23, 6.11)	0.035^*^
6 months	67.95 ± 6.428	86.6 ± 1.792	-18.65 (-21.13, -16.17)	<0.001^*^

Comparing the VAS scores between groups using independent samples t-tests showed significant differences at all times after the intervention (Table 6). While the scores at the start were similar (p = 0.104), the TF group showed much better pain relief at 1 month (t = -7.48, p < 0.001) and 3 months (t = -3.78, p < 0.001), with large effect sizes (Cohen's d: -1.93 and -0.98, respectively). While the initial pain scores were similar (p = 0.104), the TF group showed much better pain relief at 1 month (t = -7.48, p < 0.001) and 3 months (t = -3.78, p < 0.001), with large effect sizes (Cohen's d: -1.93 and -0.98, respectively). However, by 6 months, the caudal group had significantly better pain results (t = 5.09, p < 0.001), with a large effect size (d = 1.31), showing that this change in effectiveness over time is very important. Conversely, by 6 months, the caudal group exhibited significantly better pain outcomes (t = 5.09, p < 0.001), with a large effect size (d = 1.31), indicating substantial clinical significance in this temporal reversal of comparative efficacy.

**Table 6:** Statistical analysis of Visual Analog Scale (VAS) scores between transforaminal and caudal epidural steroid injection groups (N = 60) Negative effect sizes indicate lower VAS scores (less pain) in the transforaminal group; positive effect sizes indicate lower VAS scores in the caudal group; ^*^statistically significant at p < 0.05

Variable	t	df	p	Cohen's d	95% CI for Effect Size
VAS - Baseline	1.65	58	0.104	0.43	(-0.10, 0.95)
VAS - 1 month	-7.48	58	<0.001^*^	-1.93	(-2.56, -1.31)
VAS - 3 months	-3.78	58	<0.001^*^	-0.98	(-1.52, -0.43)
VAS - 6 months	5.09	58	<0.001^*^	1.31	(0.74, 1.88)

Analysis of MODI scores demonstrated significant between-group differences at all post-intervention assessments (Table 7). While baseline disability levels were comparable (p = 0.209), the TF group showed significantly better functional improvement at 1 month (t = -7.19, p < 0.001), with a large effect size (d = -1.86). However, by 3 months, the caudal group demonstrated significantly superior functional outcomes (t = 5.65, p < 0.001, d = 1.46), with this advantage strengthening substantially by 6 months (t = 10.79, p < 0.001, d = 2.79). The extraordinarily large effect size at 6 months underscores the substantial clinical significance of the caudal approach's superior long-term efficacy for reducing functional disability.

**Table 7:** Comparative statistical analysis of Modified Oswestry Disability Index (MODI) between intervention groups for lumbar radiculopathy (N = 60) Negative effect sizes indicate lower MODI scores (less disability) in the transforaminal group; positive effect sizes indicate lower MODI scores in the caudal group; ^*^statistically significant at p<0.05

Variable	t	df	p	Cohen's d	95% CI for Effect Size
MODI - Baseline	1.27	58	0.209	0.33	(-0.19, 0.85)
MODI - 1 month	-7.19	58	<0.001^*^	-1.86	(-2.48, -1.24)
MODI - 3 months	5.65	58	<0.001^*^	1.46	(0.88, 2.04)
MODI - 6 months	10.79	58	<0.001^*^	2.79	(2.06, 3.51)

Comparative analysis of CORI scores using independent t-tests revealed no significant differences at baseline (p = 0.106) or 1 month (p = 0.133), as shown in Table 8. By 3 months, the caudal group demonstrated significantly better clinical outcomes (t = 4.32, p < 0.001, d = 1.12). This advantage became substantially more pronounced at 6 months (t = 14.83, p < 0.001), with an exceptionally large effect size (d = 3.82), highlighting the marked superiority of the caudal approach for sustained clinical improvement.

**Table 8:** Statistical comparison of Clinical Outcome Rating Index (CORI) scores between different epidural approaches for lumbar radiculopathy management (N = 60) Negative effect sizes indicate lower CORI scores (better outcomes) in the transforaminal group; positive effect sizes indicate lower CORI scores in the caudal group; ^*^statistically significant at p < 0.05

Variable	t	df	p	Cohen's d	95% CI for Effect Size
CORI - Baseline	1.64	58	0.106	0.42	(-0.10, 0.95)
CORI - 1 month	-1.53	58	0.133	-0.39	(-0.91, 0.12)
CORI - 3 months	4.32	58	<0.001^*^	1.12	(0.56, 1.67)
CORI - 6 months	14.83	58	<0.001^*^	3.82	(2.98, 4.67)

Between-group comparison of MRM scores revealed statistically significant differences at all assessment timepoints (Table 9). The initial scores were significantly different (t = 2.42, p = 0.019, d = 0.63), which means the groups were not the same before the intervention, and this needs to be taken into account when interpreting the results. The TF group demonstrated markedly superior early motor recovery at 1 month (t = 12.26, p < 0.001), with a very large effect size (d = 3.17). This advantage persisted but diminished substantially by 3 months (t = 2.16, p = 0.035, d = 0.56). At 6 months, there was a significant change, with the caudal group showing much better long-term motor recovery, indicating a very strong and important effect.

**Table 9:** Temporal statistical analysis of Modified Roland-Morris (MRM) scores in lumbar radiculopathy treatment groups (N = 60) Positive effect sizes indicate higher MRM scores (better recovery) in the transforaminal group; negative effect sizes indicate higher MRM scores in the caudal group; ^*^statistically significant at p < 0.05

Variable	t	df	p	Cohen's d	95% CI for Effect Size
MRM - Baseline	2.42	58	0.019^*^	0.63	(0.10, 1.16)
MRM - 1 month	12.26	58	<0.001^*^	3.17	(2.39, 3.94)
MRM - 3 months	2.16	58	0.035^*^	0.56	(0.03, 1.09)
MRM - 6 months	-15.31	58	<0.001^*^	-3.95	(-4.84, -3.07)

Repeated measures ANOVA for VAS scores demonstrated a significant main effect of time (F = 1227.5, p < 0.001, partial η² = 0.955), indicating substantial overall pain reduction across follow-up periods, with 95.5% of variance in pain scores explained by temporal progression (Table 10). Critically, a significant time × group interaction effect was observed (F = 49.2, p < 0.001, partial η² = 0.459), confirming that pain reduction trajectories differed significantly between intervention groups, with this interaction explaining 45.9% of variance in temporal pain patterns. Post hoc Bonferroni-adjusted pairwise comparisons confirmed significant differences between all sequential timepoints (p < 0.001), supporting continuous improvement throughout the follow-up period.

**Table 10:** Repeated measures ANOVA for Visual Analog Scale Scores (VAS) in patients with lumbar radiculopathy (N = 60) ^*^statistically significant at p < 0.05; partial η² represents proportion of variance explained by each effect

Effect	Sphericity Correction	Sum of Squares	df	Mean Square	F	p	Partial η²
Time	Greenhouse-Geisser	810.2	2.60	311.15	1227.5	<0.001^*^	0.955
Time × Group	Greenhouse-Geisser	32.5	2.60	12.47	49.2	<0.001^*^	0.459
Residual	Greenhouse-Geisser	38.3	151.03	0.25	-	-	-

Longitudinal analysis of MODI scores revealed a significant main effect of time (F = 1677.0, p < 0.001, partial η² = 0.967), indicating substantial functional improvement across the follow-up period, with time explaining 96.7% of variance in disability scores (Table 11). The significant time × group interaction (F = 59.2, p < 0.001, partial η² = 0.505) confirms differential recovery trajectories between interventions, with this interaction explaining 50.5% of the variance in temporal disability patterns. Post hoc analysis with Bonferroni correction demonstrated significant differences between all timepoints for both groups (p < 0.001), confirming continuous functional improvement throughout the assessment period.

**Table 11:** Repeated measures ANOVA for Modified Oswestry Disability Index scores in epidural steroid injection treatment groups (N = 60) ^*^statistically significant at p < 0.05; partial η² represents proportion of variance explained by each effect

Effect	Sphericity Correction	Sum of Squares	df	Mean Square	F	p	Partial η²
Time	Greenhouse-Geisser	58896.2	2.11	27942.21	1677.0	<0.001^*^	0.967
Time × Group	Greenhouse-Geisser	2079.4	2.11	986.55	59.2	<0.001^*^	0.505
Residual	Greenhouse-Geisser	2036.9	122.25	16.66	-	-	-

Repeated measures ANOVA for CORI scores demonstrated a significant main effect of time (F = 22.5, p < 0.001, partial η² = 0.280), indicating overall clinical improvement across assessment periods, though with more modest variance explanation (28.0%) compared to other outcome measures (Table 12). The significant time × group interaction (F = 2.7, p < 0.001, partial η² = 0.044) confirms differential clinical outcome trajectories between interventions, albeit with a smaller effect size. Notably, Greenhouse-Geisser correction yielded substantially reduced degrees of freedom (df = 1.03), suggesting severe sphericity violation and necessitating cautious interpretation.

**Table 12:** Repeated measures ANOVA for Clinical Outcome Rating Index scores between caudal and transforaminal approaches in lumbar radiculopathy management (N= 60) *statistically significant at p < 0.05; partial η² represents proportion of variance explained by each effect

Effect	Sphericity Correction	Sum of Squares	df	Mean Square	F	p	Partial η²
Time	Greenhouse-Geisser	619.3	1.03	603.24	22.5	<0.001^*^	0.280
Time × Group	Greenhouse-Geisser	74.0	1.03	72.04	2.7	<0.001^*^	0.044
Residual	Greenhouse-Geisser	1597.2	59.55	26.82	-	-	-

Longitudinal analysis of MRM scores revealed a significant main effect of time (F = 1158.9, p < 0.001, partial η² = 0.952), indicating substantial motor recovery improvement across the follow-up period, with temporal progression explaining 95.2% of variance (Table 13). Importantly, a highly significant time × group interaction was observed (F = 173.8, p < 0.001, partial η² = 0.750), confirming markedly different recovery trajectories between interventions, with this interaction explaining 75.0% of variance in temporal motor recovery patterns - the strongest interaction effect across all outcome measures. Post hoc Bonferroni-adjusted comparisons demonstrated significant differences between all timepoints for both groups (p < 0.001), supporting continuous motor improvement throughout the assessment period.

**Table 13:** Repeated measures ANOVA for Modified Roland-Morris Scores in lumbar radiculopathy patients undergoing different epidural steroid injection techniques (N = 60) ^*^statistically significant at p < 0.05; partial η² represents proportion of variance explained by each effect

Effect	Sphericity Correction	Sum of Squares	df	Mean Square	F	p	Partial η²
Time	Greenhouse-Geisser	104044.8	2.08	50114.27	1158.9	<0.001^*^	0.952
Time × Group	Greenhouse-Geisser	15600.7	2.08	7514.25	173.8	<0.001^*^	0.750
Residual	Greenhouse-Geisser	5207.1	120.42	43.24	-	-	-

## Discussion

This study was conducted to evaluate and compare the effectiveness of Transforaminal Epidural Steroid Injections (TFESI) and Caudal Epidural Steroid Injections (CESI) in patients with lumbar radiculopathy. The primary endpoints were pain relief, functional improvement, clinical outcomes, and motor recovery, assessed over a six-month period. The results demonstrate that both approaches provide significant benefits; however, distinct differences were observed concerning the timing and duration of therapeutic effects.

Early pain relief at the one-month follow-up was notably superior in the TFESI group compared to CESI. These observations align with previous literature, which suggests that TFESI provides rapid relief due to targeted delivery directly adjacent to the inflamed nerve roots, optimizing the anti-inflammatory effect precisely where it is most needed [1,5]. Studies by Vad et al. (2002) [3] and Ackerman and Ahmad (2007) [1] support this notion, indicating rapid onset analgesia through precise localization and lower medication volume requirements, thus providing rapid symptomatic control.

However, in contrast to short-term outcomes, CESI demonstrated significantly superior long-term efficacy. At the six-month follow-up, CESI showed better improvements across all measured parameters - pain (VAS), functional capacity (MODI), clinical outcomes (CORI), and motor recovery (MRM). This finding is consistent with several other studies, including those conducted by Garg et al. (2022) and Kale et al. (2024), who reported sustained symptom relief and functional recovery with caudal epidural injections [5,6]. The broader epidural space coverage and larger medication volumes typical of CESI are likely responsible for this prolonged therapeutic benefit, addressing inflammatory processes over a wider anatomical distribution and offering durable relief [13].

From a functional perspective, the Modified Oswestry Disability Index (MODI) revealed initial superior performance with TFESI, attributable to direct nerve root targeting. Nevertheless, the CESI approach showed notably greater long-term improvement, emphasizing that the broader anti-inflammatory coverage may confer a more sustained functional advantage. This finding underscores the importance of addressing widespread inflammatory changes within the epidural space that might contribute to chronic symptomatology in lumbar radiculopathy [2,10].

Clinical outcomes assessed via CORI scores further supported the advantage of CESI, particularly evident in sustained improvements at six months. These results suggest that CESI may enhance overall patient satisfaction, reduce symptom recurrence rates, and potentially decrease the need for repeated healthcare visits and additional interventions. These implications are important for both patient quality of life and healthcare resource management, particularly given the high prevalence of lumbar radiculopathy and associated economic burdens [20,21].

Motor recovery, a crucial factor affecting patients' daily activities and overall quality of life, was notably superior in the CESI group at the six-month mark. The substantial difference in Motor Recovery Measures (MRM) could reflect better overall control of chronic inflammatory states affecting nerve root function, contributing to improved nerve health and restoration of neuromuscular capabilities. Previous literature corroborates this benefit, suggesting that CESI may offer more comprehensive neuromuscular recovery due to its broader inflammatory control [22,23].

Our findings regarding the long-term superiority of CESI align with the meta-analysis conducted by Lee et al. (2018), which reported comparable immediate effects between approaches but suggested potentially superior long-term outcomes with CESI [21]. Similarly, Liu et al. (2016) conducted a systematic review comparing transforaminal versus caudal routes for epidural steroid injections and concluded that while both approaches were effective, CESI demonstrated better sustained improvement in functional outcomes at 6 months [20].

The mechanisms underlying the differential effectiveness between TFESI and CESI have been explored by several authors. Tachihara et al. (2008) reported that the anti-inflammatory effects of steroids play a crucial role in mitigating radicular symptoms, with broader epidural distribution potentially addressing more diffuse inflammatory processes [24]. Additionally, Cuellar et al. (2010) demonstrated that inflammatory cytokines present in the epidural space contribute significantly to radicular pain, suggesting that the more extensive spread of steroid solution with CESI may better address multifactorial inflammatory mechanisms compared to the more targeted TFESI approach [16].

Strengths of the study

The primary strength of this study lies in its longitudinal design with multiple follow-up points (1, 3, and 6 months), enabling comprehensive temporal assessment of therapeutic trajectories for both interventions. The inclusion of multiple validated outcome measures (VAS, MODI, CORI, and MRM) provides a multidimensional evaluation of treatment efficacy beyond simple pain assessment, offering insights into functional recovery and disability reduction. Additionally, the study employed rigorous statistical methods, including both between-group comparisons and within-subject analyses using repeated measures ANOVA, allowing robust assessment of both immediate and sustained effects. Furthermore, the study design incorporated blinded outcome assessment to minimize observer bias, enhancing the reliability of the reported findings. Finally, the comparable baseline characteristics between groups reduced potential confounding variables, strengthening the validity of the comparative outcomes observed.

Limitations

This study has several limitations that warrant consideration when interpreting the results. Firstly, the retrospective design inherently introduces potential selection and information biases, as treatment allocation was not randomized but based on clinician discretion and patient factors. This may have resulted in systematic differences between groups despite similar baseline characteristics. Secondly, the sample size, while adequately powered for primary outcomes, may be insufficient for subgroup analyses that could identify patient-specific factors predicting differential responses to either intervention. Thirdly, the single-center nature of the study limits its generalizability to broader populations and different clinical settings. Additionally, we could not control for potential co-interventions such as physical therapy, medication use, or lifestyle modifications during the follow-up period, which might have influenced outcomes. The study also lacked a placebo or sham injection control group, making it difficult to distinguish specific treatment effects from placebo effects or the natural history of the condition. Furthermore, the assessment of outcomes was limited to 6 months, preventing evaluation of longer-term efficacy. Finally, we did not include cost-effectiveness analysis or patient satisfaction measures, which would provide valuable additional perspectives on the relative merits of each intervention in clinical practice. Future studies should address these limitations to strengthen the evidence base.

Recommendations

Future research should incorporate prospective randomized controlled designs with larger sample sizes and extended follow-up periods beyond six months to establish long-term efficacy. Studies should include detailed subgroup analyses to identify patient characteristics (such as specific radiological findings, duration of symptoms, or pain mechanisms) that might predict optimal responses to either intervention. Incorporation of cost-effectiveness analyses would provide valuable information for healthcare resource allocation. Additionally, exploration of combined approaches, modified injection techniques, or varied medication compositions might yield enhanced therapeutic protocols. Finally, neurophysiological studies examining the precise mechanisms underlying differential efficacy between the two approaches would advance our understanding of optimal intervention strategies for lumbar radiculopathy.

## Conclusions

Both transforaminal and caudal epidural steroid injections effectively alleviate symptoms associated with lumbar radiculopathy, but with distinct temporal efficacy profiles. TFESI demonstrates superior short-term pain relief and functional improvement, likely due to the precise delivery of medication to affected nerve roots. Conversely, CESI provides significantly better long-term outcomes across all parameters, suggesting broader and more sustained anti-inflammatory effects. Given its technical simplicity, favorable safety profile, and superior long-term efficacy, CESI emerges as the preferable approach for the comprehensive management of lumbar radiculopathy, particularly when sustained symptom control is prioritized. However, TFESI remains valuable for immediate symptom alleviation in acute presentations. Therapeutic decision-making should therefore consider both the temporal needs and specific clinical presentation of each patient. These findings contribute significantly to the evidence base guiding clinical practice in the non-surgical management of lumbar radiculopathy.
